# Methicillin-resistant *Staphylococcal* periprosthetic joint infections can be effectively controlled by systemic and local daptomycin

**DOI:** 10.1186/s12879-016-1366-9

**Published:** 2016-02-01

**Authors:** Feng-Chih Kuo, Shih-Hsiang Yen, Kuo-Ti Peng, Jun-Wen Wang, Mel S. Lee

**Affiliations:** 1 Department of Orthopaedic Surgery, Kaohsiung Chang Gung Memorial Hospital, No 123, Ta Pei Road, Niao Sung Dist, Kaohsiung, Taiwan; 2 Chang Gung University College of Medicine, Kaohsiung, Taiwan; 3 Department of Orthopaedic Surgery, Chiayi Chang Gung Memorial Hospital, Chiayi, Taiwan

**Keywords:** Daptomycin, Periprosthetic joint infection, Cement, *Staphylococcus*

## Abstract

**Background:**

Methicillin-resistant *Staphylococcus* remains a serious problem in the treatment of periprosthetic joint infection (PJI). Higher failure rates were reported when vancomycin was used in 2-stage exchange arthroplasty. Therefore a better therapeutic drug is needed to treat PJI caused by methicillin-resistant organisms. The purpose of the study was to evaluate the safety and efficacy of daptomycin when administered in bone cement combined with systemic use for methicillin-resistant *Staphylococci* PJI.

**Methods:**

We conducted a retrospective study from January 2010 to December 2012. Twenty-two patients (10 knees and 12 hips) with PJI caused by methicillin-resistant *Staphylococcus* species underwent 2-stage revision arthroplasty. In the first stage, 10 % daptomycin (weight daptomycin per weight bone cement) was incorporated into polymethylmethacrylate bone cement, and systemic daptomycin (6 mg/kg) was administered postoperatively for 14 days. In the second stage, 2.5 % w/w daptomycin was used in the bone cement. The minimum follow-up was 2 years or until recurrence of infection.

**Results:**

The infecting organisms included methicillin-resistant *Staphylococcus aureus* in 10 patients, methicillin-resistant *Staphylococcus epidermidis* in 8 patients and methicillin-resistant *coagulase-negative Staphylococci* in 4 patients. The mean follow-up duration was 33.7 months (range, 24–51 months). The treatment success rate was 100 %. Only one patient developed asymptomatic transient elevation of the creatine phosphokinase level. No patient experienced any adverse effects related to daptomycin such as myositis, rhabdomyolysis, peripheral neuropathy, derangement of liver function, or eosinophilic pneumonia.

**Conclusions:**

In this series, no serious adverse events occurred. Our protocol, using daptomycin-impregnated cement combined with short duration of systemic daptomycin, appears to be an effective and safe treatment for methicillin-resistant *Staphylococcus* PJI.

## Background

Periprosthetic joint infections (PJI) of the hip and knee are disastrous complications that occur in approximately 1 to 2 % of patients after total joint arthroplasties [1]. The management of PJI may require long-term antibiotic suppression, surgical debridement, one-stage or 2-stage revision, resection arthroplasty, arthrodesis, or amputation. The “gold standard” treatment for chronic PJI in North America is 2-stage revision [2]. The procedure consists of removal of the infected prosthesis in the first stage, followed by replacing it with a high-dose antibiotic cement spacer to eradicate the infection and prevent joint space contracture between stages [3]. Once the infection has been treated with systemic antibiotics, the second stage is performed to implant a new prosthesis.

Most PJI are caused by Gram-positive cocci, including *Staphylococcus* species [4]. Methicillin-resistant organisms account for up to 74 % of PJI in some reports [5]. Vancomycin is most commonly incorporated into polymethylmethacrylate (PMMA) bone cement and subsequently used intravenously for the treatment of methicillin-resistant *Staphylococcus aureus* (MRSA) [6]. The successful clinical control of chronic PJI due to methicillin-resistant organisms varies from 48 to 89 % [7, 8] in the hip and 60 to 74 % [9, 10] in the knee when vancomycin is used in 2-stage exchange arthroplasty. These results have led orthopedic surgeons to seek new therapeutic strategies for PJI caused by methicillin-resistant *Staphylococcus* spp.

Daptomycin is a novel cyclic lipopeptide antibiotic secreted by *Streptomyces roseosporus.* Daptomycin has excellent activity against Gram-positive bacteria through disruption of multiple bacterial plasma membrane functions, without penetrating the cytoplasm [11]. Clinical experience with daptomycin treatment of PJI is limited to systemic intravenous use in small case series [12–14]. Several in vitro studies also showed that daptomycin could be locally delivered from PMMA bone cement without impairing cement strength [15, 16]. Only one clinical report showed combined use of daptomycin in bone cement and intravenously to treat chronic PJI in a 2-stage surgery [17].

The aim of this study was to review the results of daptomycin used in PMMA bone cement and systemically in 2-stage exchange surgeries for the treatment of PJI due to methicillin-resistant *Staphylococcus* species.

## Methods

### Study design and setting

This retrospective, descriptive study was conducted from January 2010 to December 2012 in a 2700-bed medical center. Consecutive adults who underwent 2-stage revision arthroplasty using local and systemic daptomycin for the treatment of PJI caused by methicillin-resistant *Staphylococcus* species were included. The use of local and systemic daptomycin for the treatment of methicillin-resistant *Staphylococcus* PJI was just done during the study period. The exclusion criteria were PJI caused by methicillin-sensitive *Staphylococcus* species, Gram-negative bacteria, *Mycobacterium tuberculosis*, fungi and polymicrobial infections. Diagnosis was confirmed by isolation of methicillin-resistant bacteria in at least 2 intraoperative cultures. All clinical data were collected retrospectively by reviewing electronic medical records. The Institutional Review Board of the Chang Gung Memorial Hospital Foundation approved the study (IRB No. 103-3637B).

### Surgical procedures and postoperative care

Patients with early prosthesis infections and acute haematogenous infections with failure of DAIR surgery (debridement, antibiotics, irrigation and prosthesis retention), or with prior implant loosening and late chronic infections, underwent two-stage revision surgery [18]. The infecting organisms included MRSA in 10 patients, methicillin-resistant *Staphylococcus epidermidis* (MRSE) in 8 patients and methicillin-resistant *coagulase-negative Staphylococci* (MRCoNS) in 4 patients. The minimum inhibitory concentrations (MICs) of vancomycin were determined as > 1.5 mg/L in all patients. Higher vancomycin MICs (>1.5 mg/L) have a higher risk of treatment failure for MRSA treated with vancomycin [19]. In the first stage, the operative procedure included removal of the implants, aggressive debridement of the joint and insertion of a high-dose, daptomycin-loaded cement spacer or beads. Twenty (91 %) of the 22 patients were treated with bead fashion of antibiotic-loaded cement. Erythrocyte sedimentation rate (ESR), C-reactive protein (CRP), complete blood count (CBC) with differential and creatine phosphokinase (CPK) were monitored weekly postoperatively. In the second stage, new prostheses were reimplanted and low-dose daptomycin-loaded cement was used if cementing fixation was needed. No patients had a joint aspiration before reimplantation. The criteria for reimplantation included a reduced ESR, return to near normal CRP, and satisfactory wound status. A closed suction drain inserted immediately after each surgery was removed 2–5 days later when the amount of daily drainage was less than 60 mL per day. The postoperative course of periprosthetic knee infection treatment consisted of a 3-day period of immobilization in a hinge-knee brace, followed by gradually continuous passive motion exercise, and protected weight-bearing activity. All the revision total hip arthroplasty were posterior approach. The postoperative course of periprosthetic hip infection treatment consisted of a 3-day period of immobilization using skin traction (2 kg) followed by protected weight-bearing activity under hip abduction and brace protection to prevent dislocation.

### Composition of bone cement

If the methicillin-resistant microorganism had been identified at the time of resection arthroplasty, the dose of daptomycin was 4 g per 40 g package of bone cement (Stryker Orthopaedics, Mahwah, New Jersey) to reach therapeutic levels in the joint fluid [20]. If the infecting microorganism could not be identified preoperatively, a combination of 4 g of daptomycin and 4 g of ceftazidime (10 % w/w for each antibiotic) per 40 g package of bone cement was used. In the second stage, the antibiotic bone cement spacer or beads were carefully removed. Intraoperative tissue samples were also taken for culture, as in the first stage. All reimplantations were performed after a 2-week antibiotic holiday without elevation of ESR and CRP. After prophylaxis with intravenous 1 g vancomycin, the patients underwent prosthesis reimplantation with 1 g daptomycin (2.5 % w/w) in a pack of 40 g bone cement [Stryker Orthopaedics, Mahwah, New Jersey] without decreasing the cement strength [15, 16] for knee or hip prosthesis fixation. For knee implantation, we usually use 1 g daptomycin in a pack of 40 g bone cement for tibia and patellar component fixation and 1 g vancomycin in another pack of bone cement for femoral component fixation. For hip reimplantation, three of 12 patients used 1 g daptomycin in a pack of 40 g bone cement for cup fixation.

### Microbiological investigation

During the first stage of the 2-stage procedure, at least 3 samples of periprosthetic tissue (synovial membranes or bone) were obtained for cultures under aerobic and anaerobic conditions. One or 2 synovial fluid samples were put into blood culture flasks to increase sensitivity for the diagnosis of PJI [21]. All the samples of synovial fluid or periprosthetic tissue were incubated for at least 14 days [22]. These patients received 14 days of intravenous daptomycin 6 mg/kg/day postoperatively.

### Definition of outcome

Two-stage reimplantation was defined as being successful if the patient had no symptom or sign of infection (no pain, swelling, erythema, warmth, wound discharge, loosening of the prosthesis), had normal CRP and ESR, and did not require reoperation (including irrigation and debridement with prosthesis retention and repeat resection) after a 2-year follow-up [23]. Treatment failure was defined as: a) death related to the infection; b) recurrence of infectious symptoms and signs; c) requiring a reoperation.

### Statistical analysis

Categorical variables were expressed as percentages and numerical data as medians and ranges. Infection control rate was calculated for patients treated with daptomycin. All patients were included in the analysis.

## Results

Seventy-six patients had PJI of the knee and hip during the study period. Twenty-two (10 knees, 12 hips) had PJI caused by methicillin-resistant *Staphylococcus* species and underwent 2-stage revision arthroplasty, with daptomycin used in PMMA bone cement and systemically (Table 1). There were 16 men and 6 women. Two patients had *Staphylococcus aureus* bacteremia at first presentation. The average age at the time of the 2-stage revision was 64.4 years (range, 38–87 years). The mean Charlson comorbidity index was 3.95 (range, 2–6). The surgical procedures before enrolment included primary hip arthroplasty (10 patients), primary knee arthroplasty (8), revision total hip arthroplasty (2) and revision total knee arthroplasty (2). Fourteen subjects underwent debridement with prosthesis retention before the 2-stage revision. The mean interval between the previous surgery and the first stage of the two-stage revision was 32 (8–120) months. The mean interim period between the two-stage debridement and reimplantation was averaged 14 weeks (range, 10–18 weeks). There was no breakage of the cement spacer during the interim. The mean follow-up duration was 33.7 months (range, 24–57 months). No patient was lost to follow-up. The treatment success rate was 100 % without recurrence of infection. One patient developed asymptomatic transient elevation of the CPK level. No adverse effect related to daptomycin, such as myositis, rhabdomyolysis, peripheral neuropathy, derangement of liver function or eosinophilic pneumonia was observed in our study.

**Table 1:** Characteristics of patients with periprosthetic joint infections caused by methicillin-resistant *Staphylococcus* undergoing 2-stage revision arthroplasties

No.	Sex	Age	Surgical procedures	CCI	Pathogen	Antibiotic regimen (bone cement g/systemic mg/kg)	Use daptomycin in bone cement at reimplantation (Y/N)	FU (months)	Outcome
1	M	65	DAIR for TKA	4	MRSA	DAP 4/DAP 6	Y	26	Infection controlled
2	M	51	THA	4	MRSA	DAP 4 + CEF 4/DAP 6	N	32	Infection controlled
3	M	72	DAIR for revision TKA	5	MRSE	DAP 4/DAP 6	Y	43	Infection controlled
4	M	53	Revision THA	2	MRCoNS	DAP 4 + CEF 4/DAP 6	N	42	Infection controlled
5	F	60	TKA	3	MRSA	DAP 4 + CEF 4/DAP 6	Y	38	Infection controlled
6	F	80	DAIR for TKA	5	MRSE	DAP 4/DAP 6	Y	26	Infection controlled
7	M	66	DAIR for THA	3	MRCoNS	DAP 4/DAP 6	N	51	Infection controlled
8	M	44	DAIR for THA	2	MRSE	DAP 4/DAP 6	N	42	Infection controlled
9	M	74	DAIR for TKA	4	MRSE	DAP 4/DAP 6	Y	25	Infection controlled
10	M	45	DAIR for THA	3	MRSE	DAP 4/DAP 6	N	26	Infection controlled
11	M	51	DAIR for THA	4	MRSA	DAP 4/DAP 6	Y	44	Infection controlled
12	F	67	TKA	5	MRSA	DAP 4 + CEF 4/DAP 6	Y	32	Infection controlled
13	F	86	TKA	5	MRSA	DAP 4 + CEF 4/DAP 6	Y	37	Infection controlled
14	M	51	DAIR for THA	5	MRSA	DAP 4/DAP 6	N	40	Infection controlled
15	F	86	TKA	5	MRSA	DAP 4 + CEF 4/DAP 6	Y	30	Infection controlled
16	F	81	THA	3	MRSA	DAP 4 + CEF 4/DAP 6	Y	32	Infection controlled
17	M	81	DAIR for revision TKA	5	MRSE	DAP 4/DAP 6	Y	30	Infection controlled
18	M	51	DAIR for revision THA	4	MRCoNS	DAP 4/DAP 6	N	31	Infection controlled
19	M	50	DAIR for THA	2	MRSE	DAP 4/DAP 6	N	27	Infection controlled
20	M	87	TKA	6	MRSA	DAP 4 + CEF 4/DAP 6	Y	24	Infection controlled
21	M	38	DAIR for THA	2	MRSE	DAP 4/DAP 6	N	30	Infection controlled
22	M	78	DAIR for revision THA	6	MRCoNS	DAP 4/DAP 6	Y	33	Infection controlled

*DAIR* debridement, antibiotics, irrigation and prosthesis retention, *CCI* Charlson comorbidity index, *DM* diabetes mellitus, *HTN* hypertension, *CVA* cerebral vascular accident, *TKA* total knee arthroplasty, *LC* liver cirrhosis, *RA* rheumatoid arthritis, *THA* total hip arthroplasty, *CAD* coronary artery disease, *BMI* body mass index, *HBV* hepatic B virus, *HCV* hepatic C virus, *CKD* chronic kidney disease, *COPD* chronic obstructive pulmonary disease, *ESRD* end-stage renal disease, *DAP* daptomycin, *CEF* ceftazidime, *MRSA* methicillin-resistant *Staphylococcus aureus*, *MRCoNS* methicillin-resistant coagulase-negative *Staphylococci*, *MRSE* methicillin-resistant *Staphylococcus epidermis*, *FU* follow-up

## Discussion

Methicillin-resistant *Staphylococci* remain a challenge because the current protocol often has inferior results compared with protocols used with other organisms for the treatment of PJI [8, 24, 25]. The 2-stage protocol for resistant organisms has had success rates ranging between 48 and 89 % [7–10, 24, 25], while the average success rate for less virulent organisms was 85 % ~ 95 % [3, 8]. Most protocols used vancomycin and an aminoglycoside in PMMA bone cement combined with systemic vancomycin for 2 ~ 6 weeks after first-stage resection arthroplasty [8–10, 26]. To our knowledge, this study was the first case-series report of daptomycin used in PMMA bone cement to treat PJI caused solely by methicillin-resistant *Staphylococcus* species. Using 4 gm daptomycin in 40-g PMMA bone cement combined with subsequent systemic use for 14 days in the first stage, followed by 1 g daptomycin in 40-g PMMA bone cement in the second stage, we achieved a 100 % infection control rate with a mean follow-up of 2.8 years. We attributed the results to the readiness of daptomycin release from the PMMA bone cement and its excellent bactericidal effect against methicillin-resistant strains. Hall et al. showed that daptomycin could be released from PMMA cement at a rate similar to that of vancomycin in vitro study [27]. These results suggested that local concentrations of daptomycin from daptomycin-loaded PMMA cement were above the MIC value for most Gram-positive cocci. The efficacy of daptomycin against *Staphylococci*, compared to vancomycin, has been demonstrated in vivo and in vitro studies. Daptomycin had a higher bactericidal rate than vancomycin (92 % vs. 70 %) in an in vitro study [28]. Daptomycin showed greater and more rapid bactericidal activity than vancomycin in mice infected by MRSA [29]. Systemic daptomycin also had a higher success rate than vancomycin in 2-stage revision arthroplasty in a randomized controlled trial [14].

The actual dosage of daptomycin added in the bone cement as a spacer to treat PJI is unclear. Cortes et al. reported the first case report of use of daptomycin 10 g and gentamicin 10 g in the bone cement (each agent at 5 % w/w) in two-stage revision hip surgery for prosthetic joint infection [17]. In an international consensus meeting on periprosthetic joint infection, 2 g daptomycin (5 % w/w) was recommended in spacers [30]. Rouse et al. found that 3 g daptomycin (7.5 % w/w) did not decrease the tensile or compressive strength of PMMA bone cement and retained biologic activity after PMMA cement polymerization in an in vivo rat model [31]. According to an in vitro study, the mean percentage of daptomycin elution increased with an increase in daptomycin loading [2.5, 7.5, and 15.0 % w/w] in PMMA bone cement. Therefore, we thought higher dose daptomycin (10 % w/w) in PMMA bone cement to reach local therapeutic levels for the treatment of PJI due to methicillin-resistant *Staphylococcus* species.

The safety of systemic use of daptomycin after resection arthroplasty is also a concern. Byren et al. [14] used daptomycin at 6 or 8 mg/kg for 6 weeks after prosthesis removal in a randomized trial. They found 8 % (2 of 25) adverse events (AEs) in the 6-mg/kg group and 16.7 % (4 of 24) AEs in the 8-mg/kg group. The AEs included skin rash, rhabdomyolysis and increase CPK. In another study, severe side effects (one case of acute renal failure due to massive rhabdomyolysis, one of eosinophilic pneumonia and 2 cases of asymptomatic transient CPK level elevation) were also reported with daptomycin at a dose of 6.6 mg/kg/day for an average of 44.9 days in the treatment of PJI [13]. Our group underwent 2-week systemic antibiotic therapy after first-stage surgery without obviously poorer results than in other reports [32]. By decreasing the duration of systemic daptomycin use, 21 of 22 patients tolerated the treatment. No patients developed gastrointestinal or food intolerance. Only one patient developed asymptomatic transient elevation of the CPK level. No patient experienced any severe adverse effects related to daptomycin. Daptomycin also possesses clinical and practical advantage over vancomycin, like once daily versus twice daily dosing, less therapeutic drug monitoring and potential cost savings. We thought a shorter course of systemic use of daptomycin would be advisable because the adverse events would be fewer and the infection control rates would not be compromised.

In this study, we had used prophylactic intravenous vancomycin combined with local daptomycin for the treatment of MRSA PJI at reimplantaiton stage. Vancomycin has been known as the drug of choice to prevent MRSA PJI in primary or reimplantation arthroplasties following MRSA infection [33, 34]. We did not choose intravenous daptomycin for 2 reasons. First, we would like to compare this result with our previous experience of using systemic and local vancomycin by changing the topical antibiotic regime only. This could reduce the confounding effect if systemic daptomycin were used. Second, our infection control policy precluded us to supersede the first-line vancomycin to the second-line daptomycin for systemic use without drug sensitivity test and minimal inhibition concentration test.

The study has limitations. First, it was a retrospective design, and we could not know what proportion of patients would fail the two-stage protocol if vancomycin were used. The evaluation of a prospective cohort comparing daptomycin to vancomycin may be warranted in the future. Second, the sample size was small, making it difficult to obtain statistically significant results. Third, the optimal ratio of daptomycin to PMMA cement in vivo study was unknown, and we did not check the daptomycin level in the joint fluid. The strength of our study is that it offered clinical data from a cohort of patients, and reported the safe use of daptomycin in PMMA cement and intravenously in 2-stage revision surgery. But further studies are required to evaluate the long-term outcomes.

## Conclusions

In conclusion, daptomycin-impregnated cement combined with a short duration of systemic daptomycin appears to be an effective treatment for methicillin-resistant *Staphylococc*i PJI. The protocol could lessen the AEs related to daptomycin and provided satisfactory infection control rates.
